# Influenza A(H5N1) detection in two asymptomatic poultry farm workers in Spain, September to October 2022: suspected environmental contamination

**DOI:** 10.2807/1560-7917.ES.2023.28.8.2300107

**Published:** 2023-02-23

**Authors:** Esteban Aznar, Inmaculada Casas, Alejandro González Praetorius, María José Ruano Ramos, Francisco Pozo, María José Sierra Moros, María Victoria García Rivera, Azucena Sánchez Sánchez, Elena García Villacieros, Gabriela Saravia, María Iglesias-Caballero, Elena Román Marcos, Lucía García San Miguel

**Affiliations:** 1 Coordinating Centre for Health Alerts and Emergencies, Ministry of Health, Madrid, Spain; 2 Influenza and Respiratory Unit, National Centre for Microbiology, Instituto de Salud Carlos III, Madrid, Spain; 3 Microbiology laboratory, Hospital General Universitario de Guadalajara, Guadalajara, Spain; 4 Laboratorio Central de Veterinaria, Ministerio de Agricultura, Pesca y Alimentación, Algete, Spain; 5 CIBER in Infectious Diseases (CIBERINFEC), Madrid, Spain; 6 Consejería de Sanidad de la Junta de Castilla-La Mancha, Dirección General de Salud Pública, Departamento de Epidemiología, Toledo, Spain; 7 Subdirección General de Sanidad e Higiene Animal y Trazabilidad, Ministerio de Agricultura, Pesca y Alimentación, Madrid, Spain

**Keywords:** highly pathogenic avian influenza, HPAI A(H5N1), Spain, human, asymptomatic, outbreak

## Abstract

In autumn 2022, the Spanish Influenza National Reference Laboratory (NRL) confirmed the detection of influenza A(H5N1) in samples from two asymptomatic workers linked to an outbreak in a poultry farm in Spain. Nasopharyngeal swabs were taken according to a national screening protocol for exposed workers. Absence of symptoms, low viral load and negative serology in both workers suggested environmental contamination. These findings motivated an update of the early detection strategy specifying timing and sampling conditions in asymptomatic exposed persons.

The increase in the number of highly pathogenic avian influenza (HPAI) A(H5N1) outbreaks in birds (wild birds, poultry and domestic birds) and the indications of a possible increase in transmissibility in mammals in the 2021/22 northern hemisphere influenza season and at the beginning of the 2022/23 season have generated greater concern about the possible appearance of cases in humans [1]. We report the detection of influenza A(H5N1) in two asymptomatic workers linked to an outbreak of HPAI A(H5N1) in a poultry farm in the autumn of 2022 in Spain. Interpretation of these findings and implications in surveillance strategies are discussed.

## Event detection and description

On 20 September 2022, an outbreak of HPAI A(H5N1) was confirmed in a poultry farm in the autonomous region of Castilla-La Mancha, Spain [2]. Two days later, screening for influenza was performed on all 12 exposed workers. A nasopharyngeal swab from one worker, aged ca 20 years who was asymptomatic, was positive for influenza A by RT-PCR performed at the regional reference laboratory on 22 September. The presence of influenza A(H5N1) was confirmed by PCR by the Influenza National Reference Laboratory (NRL) on 27 September. After notification to the World Health Organization (WHO) on 4 October in accordance with International Health Regulation (IHR), a respiratory sample and an additional serum sample taken on 8 October were sent to the WHO Reference Laboratory (WRL) in London, United Kingdom yielding negative results for PCR detection and serology.

In response to the outbreak, culling of all hens at the farm (19,206 were culled and 130,941 died of the infection) was completed by 13 October and a new screening for influenza was conducted in all workers. This screening involved the initial 12 workers and 14 others who were involved in control tasks. A second asymptomatic worker in their late 20s tested positive for influenza A(H5N1) at the NRL. Samples for serology from this second worker were also taken on 19 October and 23 November. They were sent to the WRL and yielded a negative result.

Both workers were involved in egg collection and routine cleaning tasks. They used personal protection equipment, including an FFP2 mask, gloves, boots and an apron. After the outbreak confirmation, they helped in removal of dead hens, cleaning and disinfection of the premises, which ended on 22 October 2022.

The asymptomatic workers stayed in self-isolation, per the national protocol, from the first RT-PCR positive result until a second, negative, sample was obtained (on day 6 and day 9 for Worker 1 and 2, respectively). The other 24 workers remained asymptomatic throughout the monitoring period (up to 10 days after the last day of exposure) and tested negative in both screenings. 

The absence of symptoms in both workers together with the laboratory results, which showed a very low viral load and the absence of specific H5 antibodies against the A/H5 virus, suggested that the positive results in the PCR were most likely due to environmental contamination. Of note, all samples (nasopharyngeal swabs) were taken outside the farm at a healthcare centre.

## Public health response and control measures

The Practical Operations Manual of the Spanish Ministry of Agriculture, Fisheries and Food establishes the containment measures to be implemented in farms where HPAI outbreaks are detected [3]. Control measures, overseen by the regional official veterinary services, included the on-site culling of the birds, the destruction of contaminated material and the cleaning and disinfection of the facilities.

Passive surveillance of symptoms and RT-PCR testing of exposed workers were implemented in accordance with the national protocol [4]. The protocol indicates the tests should be performed as soon as possible after the confirmation of the outbreak and again 5 days after the last day of exposure. However, as an extraordinary precaution after the first worker tested positive, regional public health authorities decided to perform a second screening on the same day the culling ended, while cleaning and disinfection was still ongoing.

Contact tracing identified one household contact for Worker 1 and two for Worker 2. All three contacts remained asymptomatic during the 10-day follow-up and tested negative by PCR.

Since the first detection on 27 September and up to 31 December 2022, influenza A-positive samples from all infections in humans in the same administrative area where the farm was located – 198 in total – were subtyped by RT-PCR obtaining negative results for A(H5N1).

## Virological investigation

At the NRL, influenza A was first confirmed using a real-time multiplex RT-PCR for influenza A, B and C [5], resulting positive for influenza A with low viral load (based on quantification cycle (Cq) values) of 35.62 and 33.60, respectively. Subtyping of haemagglutinin (HA) of the influenza A virus by real-time RT-PCR identified an A(H5) subtype at low viral load of 35.84 and 34.61 Cq values, respectively.

Whole genome sequences based on universal primers previously described and adapted to NRL [6], were generated from the samples obtained from the two workers and shared through GISAID [7] (Table). To increase the sequencing success given the low viral load, a NovaSeq 6000 System was used to sequence both samples. Sequences from Worker 1 were complete for all segments but for Worker 2, the sequencing of the polymerase genes failed but segments HA, partial NP, NA, MP and NS were obtained (Table).

**Table:** GISAID accession numbers of sequences from the two farm workers and two reference laying hens affected in the highly pathogenic avian influenza A(H5N1) outbreak, Spain, 20 September–13 October 2022

Sample	Isolate name	Isolate ID	PB2 segment ID	PB1 segment ID	PA segment ID	HA segment ID	NP segment ID	NA segment ID	MP segment ID	NS segment ID
Worker 1	A/CastillaLaMancha/3739/2022	EPI_ISL_15542438	EPI2197458	EPI2197459	EPI2197457	EPI2197461	EPI2197455	EPI2197460	EPI2352712	EPI2197456
Worker 2	A/CastillaLaMancha/3869/2022	EPI_ISL_16813290	NP	NP	NP	EPI2352966	EPI2352962 (partial)	EPI2352965	EPI2352964	EPI2352963
Hen 1	A/laying_hen/Spain/3232–37_22VIR10586–4/2022	EPI_ISL_15878546	EPI2220650	EPI2220651	EPI2220649	EPI2220653	EPI2220646	EPI2220652	EPI2220648	EPI2220647
Hen 2	A/laying_hen/Spain/3232–35_22VIR10586–3/2022	EPI_ISL_15878545	EPI2220642	EPI2220643	EPI2220641	EPI2220645	EPI2220638	EPI2220644	EPI2220640	EPI2220639

NP: not possible.

Virus sequences from samples obtained from the two workers were shared through GISAID [7]. Hen sequences were submitted to the GISAID by the European Reference Laboratory for Avian Influenza.

The analysis of the complete HA gene segment showed that the HPAI H5N1 human viruses belong to clade 2.3.4.4b. Clustering of the two viral genomes from farm workers indicates that they are highly related with those obtained from sequenced laying hen genomes from the same farm. Hen sequences were submitted to the GISAID database by the European Reference laboratory (EURL) for avian influenza (Table). The sequence homology in segments PB2, PB1, PA, NA, MP and NS is 100% between human sequences and those corresponding to laying-hens. The virus from Worker 1 presented two amino acid differences in comparison with the viruses from the laying hens (HA-H289-I (H276I, H3 numbering) and NP-Q327R). The sequence from Worker 2 presented one amino acid difference (HA-I531M (I515M, H3 numbering)) from the hen samples.

## Epidemiological context

The HPAI epizootic observed in the 2021/22 season, mainly because of the A(H5N1) subtype, has been the largest recorded to date in Europe [1] and the Americas [8]. Despite this situation, only four additional detections in humans in addition to the two described here have been reported from 2021 [9].

**Figure 1:**
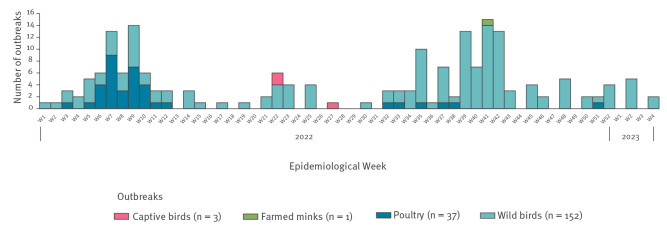
Highly pathogenic avian influenza A(H5N1) outbreaks in birds and mammals, Spain, week 1 2022−week 4 2023 (n = 193)

**Figure 2:**
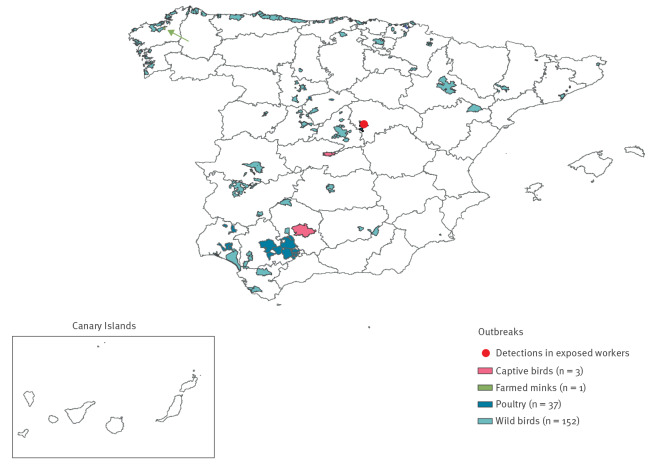
Geographical location of highly pathogenic avian influenza A(H5N1) animal outbreaks (n = 193) and human detections (n = 2) by municipality, Spain, week 1 2022−week 4 2023

Before the 2021/22 season, there had only been one detection of influenza A(H5N1) in a wild bird in Spain, which occurred in 2006 [10]. The number of HPAI outbreaks in animals in Spain since January 2022 up to week 4 2023 is presented (Figures 1 and 2).

The farmed mink outbreak (green) is also denoted by an arrow.

The HPAI epidemiological situation led to the update of the Prevention, Early Detection and Actions against Avian Influenza Protocol on 4 March 2022, in which the screening of all farm workers exposed to outbreaks was incorporated. From this update to week 4 2023, 133 exposed farm workers from 12 premises across Spain (100% of infected farms) were tested for influenza and all were negative, except for the two individuals described here.

## Discussion

Although bird to human transmission of HPAI A(H5N1) is considered unusual and person-to-person transmission is very rare, a few human cases and high death rates have been recorded in some countries since 2003 [9]. Furthermore, given their rapid evolution, influenza A viruses might eventually acquire genetic changes that enhance the ability to transmit efficiently between people. Given the changes observed in the pattern of HPAI A(H5N1) during the 2022/23 season in Europe, which extended into the summer months, it is advisable to reinforce the surveillance of possible zoonotic transmission events at the animal–human interface. In addition, using a common approach for early detection of human cases is desired so results are comparable and harmonised between countries. 

Testing of asymptomatic exposed persons can be implemented to further strengthen surveillance around HAPI outbreaks in poultry farms, although it raises the possibility of difficult interpretation of equivocal results. Currently, the European Centre for Disease Prevention and Control (ECDC) recommendations and national protocols in most of the European countries do not include PCR screening for asymptomatic HPAI-exposed persons [11-14]. Spain implemented this measure after updating the national protocol in March 2022, which has led to a more comprehensive surveillance of eventual human cases linked to outbreaks in poultry farms. However, these two positive results in asymptomatic workers raised the problem of misclassification because of possible environmental contamination. Moreover, the classification of a confirmed A(H5N1) case according to the ECDC definition [15] obligates notification under IHR. False positives have negative consequences that include social stigma, unnecessary use of pharmaceutical and non-pharmaceutical interventions, economic impact for the food industry or difficulties in risk communication to the public. To minimise the chances of contamination, public health protocols should clearly specify the right conditions and timing for swabbing. As a result of the event described, the Spanish protocol was updated with these details on 3 February 2023, stating that human cases will only be considered confirmed if samples are taken under adequate hygienic conditions, i.e. wearing clean clothes and sampling at a healthcare center rather than on location, and by swabbing 5 and 7 days after the last exposure and avoiding the sampling after the workday [16].

## Conclusions

Following the observed increase in the number of HPAI outbreaks, concern about the possible occurrence of human cases has increased. Screening of exposed workers on affected farms is a measure aimed at improving early detection of zoonotic infections. However, the appropriate conditions for carrying out the tests to avoid contamination and the criteria for interpreting the results must be considered. Biosecurity measures, including seasonal influenza vaccination of workers and the use of appropriate PPE, are key to prevent spillover on farms with detected outbreaks.
